# Segmental Volume and Circulatory Changes that Occur in Humans and Rhesus Monkeys During 4 Hour, −6 Degree Head Down Tilt

**DOI:** 10.2478/joeb-2021-0003

**Published:** 2021-11-20

**Authors:** Leslie David Montgomery, Clarence Oloff

**Affiliations:** 1 LDM Associates, San Jose, USA; 2 San Jose USA

**Keywords:** Bioimpedance, fluid redistribution, segmental volume

## Abstract

Nonhuman primates are often used to investigate physiologic processes that occur in man during aerospace/cardiovascular orthostatic research. Few studies have compared nonhuman primates and man under identical test conditions to assess the degree of similarity between the two species. Impedance plethysmography was used to measure calf, thigh, pelvic, thoracic, upper arm, and lower arm volume changes in eight rhesus (Macacca Mulatta) monkeys and twelve human subjects during four hour exposures to −6 degree head down tilt (HDT).

## Introduction

Rhesus monkeys (Macaca Mulatta) are often selected as the animal model for humans during research on the physiological effects of simulated microgravity (1). This choice is made because of their presumed similarity to man and due to the extensive amount of background information available on this species with regard to actual and simulated weightlessness. Since these animals have evolved with the Earth-gravity vector directed along the long axis, the +Gz direction, of their body, as has man, these animals are considered a more appropriate analog than subprimate quadrupeds such as pigs, dogs, cats, rabbits, or rats (2) for cardiovascular/fluid redistribution investigations. However, few studies have compared nonhuman primates and man under identical test conditions to verify the actual degree of similarity between the two species.

## Materials and methods

The specific objectives of this investigation were 1) to measure the fluid redistribution and peripheral hemodynamic changes that took place in eight unanesthetized Rhesus monkeys during antiorthostatic simulation of the initial exposure to microgravity and 2) to compare these results with those obtained earlier (3) from human subjects under similar test conditions.

The instrumentation and procedures used in this investigation were identical to those described earlier (3) for the study of human subjects during the same type of antiorthostatic stress tests. In this way, the results of the current study are directly comparable to those obtained from human subjects (3) and may be used to identify the extent to which the Rhesus monkey can be used as a substitute for man during short term antiorthostatic simulation of microgravity.

Each primate was tested for a total of approximately 6 hours; 30 minutes instrumentation, 60 minutes horizontal control period, four hour −6 degree head down tilt (HDT) test period, and a 30-minute horizontal recovery period. The U.S.A.F. Timed Rotopositioner (4) was used to restrain the animals and to provide the automated positioning and data acquisition system. Tetrapolar impedance plethysmographic (IPG) recordings were made every 30 seconds of the fluid volume of each animal's calf, thigh, pelvic, thoracic, upper arm, and lower arm body segments. Pulsatile IPG blood flow data traces were recorded continuously for 30 sec from each body segment upon initial instrumentation (Pre-Control); 4 hours after being placed in the HDT position; and 30 minutes after being returned to the horizontal position following HDT (Recovery). Systolic and diastolic blood pressures, heart rate, and rectal temperature were recorded manually every 10 minutes during the total test protocol.

Details of the experimental protocol are given below.

### A Description of impedance plethysmography

An impedance plethysmograph measures the electrical impedance changes that vary with the blood/fluid content of a body segment during each cardiac cycle or repositioning of the body. With a tetrapolar arrangement, four electrodes are placed on or around the given segment, separated by various axial distances. A high-frequency, low-amperage electrical current is applied between the outer two electrodes. Simultaneously, the resistance of the electrically conductive tissue and blood in the segment is measured between the two inner electrodes.

The measured resistance of the given body segment at any point in time is directly related to segmental length and a resistivity factor and inversely related to the average segmental cross-sectional area, which may be written:

(1) $$R=\frac{\rho \cdot L}{A}$$

where ρ is the electrical resistivity of the material in ohm-centimeters, L is the length of the body segment in centimeters, and A is the average cross-sectional area of the segment in square centimeters.

Multiplying this expression by unity in the form L/L and equating the denominator to volume, since V = AL, Eq. (1) may be written as:

(2) $$R=\frac{\rho \cdot L^{2}}{V}$$

As explained by Nyboer (5), if the length of the body segment is considered to remain constant during a short-term experimental treatment and any change in the segmental volume is considered to be caused by a change in the average cross-sectional area, then any change in segmental volume, ΔV, is proportional to a measured change in segmental resistance, ΔR. Mathematically:

$$\Delta R=R_{1}-R_{2}=\rho \cdot L^{2}\left(\frac{1}{V_{1}}-\frac{1}{V_{2}}\right)$$

or

$$\Delta R=\rho \cdot L^{2}\left(\frac{V_{2}-V_{1}}{V_{1}\cdot V_{2}}\right)=\rho \cdot L^{2}\left(\frac{\Delta V}{V_{1}\cdot V_{2}}\right)$$

which by substitution,

(3) $$\Delta R\approx -\rho \cdot L^{2}\frac{\Delta V}{V}\approx -R\frac{\Delta V}{V}$$

Thus, Equation 3 provides the relation between small changes in volume and the corresponding changes in electrical resistance of the given body segment. In this way, a fractional change in segmental volume is closely related to the fractional change in measured resistance, which may be expressed by the ratio:

(4) $$\frac{\Delta V}{V_{0}}\approx -\frac{\Delta R}{R_{0}}$$

Using Equation 4, records of segmental base resistance can be analyzed to obtain an index of the percentage change in segmental volume that takes place during various stress sequences.

**Figure 1:**
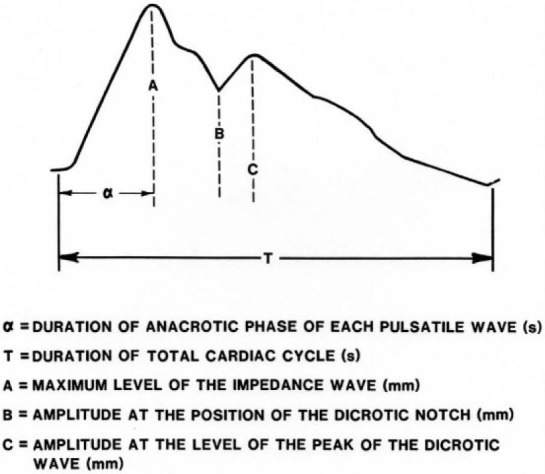
Graphical technique used to analyze the IPG waveform.

Pulsatile resistance changes that take place during each cardiac cycle may be recorded using an impedance plethysmograph and closely resemble the blood pressure pulse as shown in Figure 1. Additional blood accumulating between the two inner electrodes of a tetrapolar system during a given cardiac cycle has the equivalent electrical effect of placing a second, variable resistor in parallel with the tissue (basal) resistance lying between the two recording electrodes. This, in turn, decreases the total segmental resistance between the recording electrodes as a function of the pulse volume during the cardiac cycle. Specific dimensions obtained from the shape of the impedance pulse waveform (Figure 1) can be used to calculate indices of the elasticity and tone of each segment's vascular system as follows:

The rheographic index (RI), is a measure of the relative level of blood filling of a given body segment as judged from the maximum amplitude of the impedance pulse (A) converted to fractions of an ohm using a calibration factor = Amplitude x (calibration deflection for a known resistance value), (6).

The anacrotic index (AI), the ratio of the duration of the anacrotic phase of the pulse wave to the duration of the entire cardiac cycle (a/T), is used to assess the elasticity and tone of the vessels with large and intermediate lumens (7).

The dicrotic index (DCI) which is defined as the ratio of the amplitude of the wave at the height of the incisure to the maximum pulse amplitude (B/A), reflects primarily the tone of the arterioles (8).

The diastolic index (DSI), the ratio of the amplitude at the level of the peak of the dicrotic wave to the maximum pulse amplitude (C/A) may be used to monitor the state of blood outflow and venous tone of the segment (9).

These indices of volume and hemodynamic state were selected for use in this investigation since they can be derived from the most fundamental impedance measures and are independent of segmental resistivity, geometry, or assumed mathematical transformations. When obtained using standardized equipment and procedures they are well suited for cross specie comparative studies between human and nonhuman primates.

### B Experimental Method

#### 1 Conditions

All observations were made at an average ambient temperature of 21.0 ± 0.5 °C with the animals resting in either a horizontal or antiorthostatic (HDT) position. Air movement within the room was negligible. Bright light, excessive noise, or other vasoactive stimulation were kept to a minimum during each test sequence.

#### 2 Pulse Rate

All animals were instrumented with the standard sternal ECG leads for monitoring heart rate and rhythm periodically during each test sequence.

#### 3 Blood Pressure

Left brachial systolic and diastolic blood pressures were recorded periodically (at five minute intervals) during each phase of the study. All blood pressures were taken with a conventional automatic manometric system.

#### 4 Internal Body Temperature

Rectal temperature was measured using an YSI, Inc. 700 thermocouple probe inserted 10 cm into the rectum and held in place by means of non-constricting adhesive tape.

#### 5 Segmental Blood Flow and Volume

Measurements of baseline resistance (R_0_) and resistance changes (ΔR) were made of the calf, thigh, pelvic, torso, upper arm, and lower arm body segments prior to, during and following each test sequence using impedance plethysmography.

**Figure 2:**
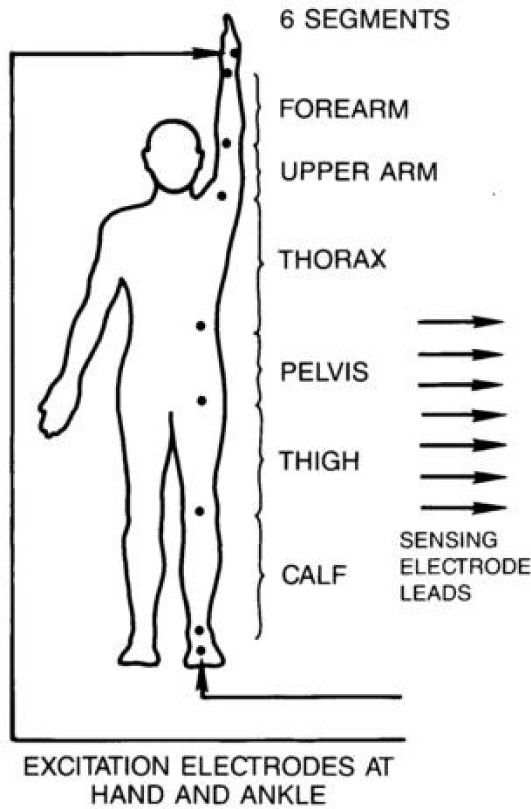
Impedance electrode locations on humans and Rhesus monkeys.

Disposable ECG electrodes were attached (as shown in Figure 2) to the foot, ankle, knee, and midthigh (opposite the groin) region of each animal's right leg; on the same side of the body at the iliac crest; and on the right arm on the back of the hand, at the wrist, elbow, and on the shoulder. A UFI Inc. tetrapolar IPG (UFI Inc., Morro Bay, CA) was used to introduce a high frequency (~50 kHz), low amperage (0.1 mA rms) constant current signal between the foot and hand electrodes. Simultaneous Ro and ΔR values were measured in each segment in sequential order periodically during each test. Records of Ro and ΔR were analyzed, as described above, to determine the fluid volume and blood flow responses of each segment to HDT.

#### 6 Anesthetic

Each animal was given an initial intra-muscular injection of 20 mg/kg ketamine prior to each tilt table test sequence to facilitate placement within the restraint system (4) and instrumentation. Each monkey was then allowed to regain and remain fully conscious prior to initiation of the control period and subsequent phases of the experiment.

### C Statistical tests used

Independent Student's t-tests were used to compare the human and Rhesus monkey volume and circulatory changes at 240 minutes elapsed time. All statistical tests were performed using the SYSTAT (10) computer program with a rejection criterion of P<0.05.

### Informed consent

Informed consent has been obtained from all individuals included in this study.

### Ethical approval

The research related to human use has been complied with all relevant national regulations, institutional policies and in accordance with the tenets of the Helsinki Declaration, and has been approved by the authors’ institutional review board or equivalent committee.

The research related to animals use has been complied with all the relevant national regulations and institutional policies for the care and use of animals.

## Results

Table 1 lists the 240 min HDT % volumes (relative to those at the start of HDT) ± S.E. for the Rhesus monkeys used in this experiment and the corresponding volumes that were found during the similar HDT study that was conducted using human subjects (3).

**Table 1:** Percent segmental volumes relative to control values after 4 hours of −6 degrees head down tilt.

GROUP	TIME	NUMBER	CAFM	CAFSE	THIM	THISE	PELM	PELSE
HUMAN	240	12	0.947	0.011	0.976	0.008	1.031	0.037
RHESUS	240	8	0.931	0.037	0.965	0.034	0.997	0.027

**GROUP**	**TIME**	**NUMBER**	**TORM**	**TORSE**	**UARMM**	**UARMSE**	**LARM**	**LARMSE**
HUMAN	240	12	1.018	0.013	0.998	0.018	0.989	0.010
RHESUS	240	8	1.040	0.015	1.043	0.022	0.970	0.025

Segment designation: CAF=calf, THI=thigh, PEL=pelvic, TOR=torso, UARM=upper arm, LARM=lower arm

Segment name - M = Group mean percent volume change from control

Segment name - SE = Group standard error

**Figure 3:**
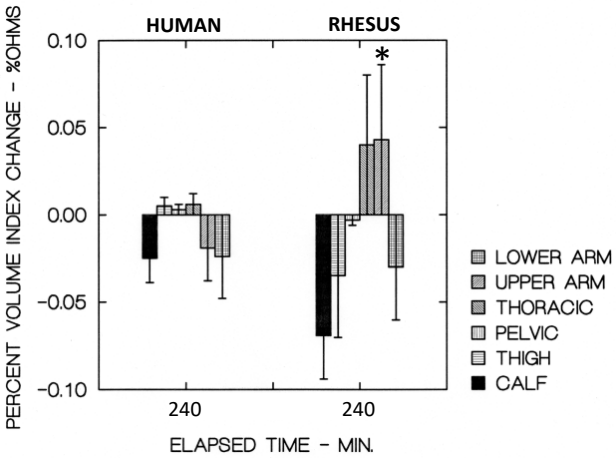
Human and Rhesus monkey % volume index change after 4 hr, −6 deg HDT. * Significantly different (P<0.05) than similar segment of human subjects

**Figure 4:**
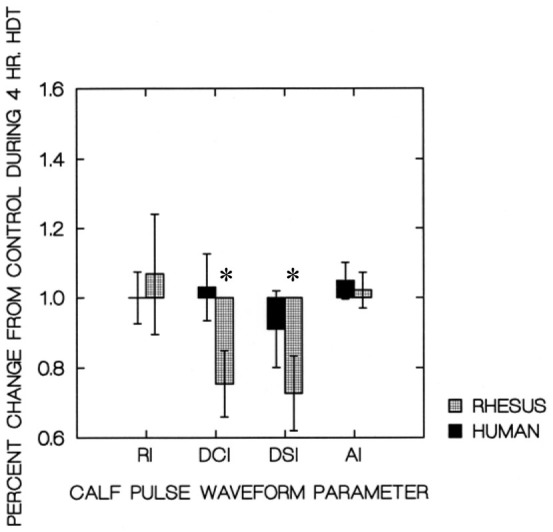
Calf Waveform parameters at 4 hr HDT. * Significantly different (P<0.05) than similar segment of human subjects.

**Figure 5:**
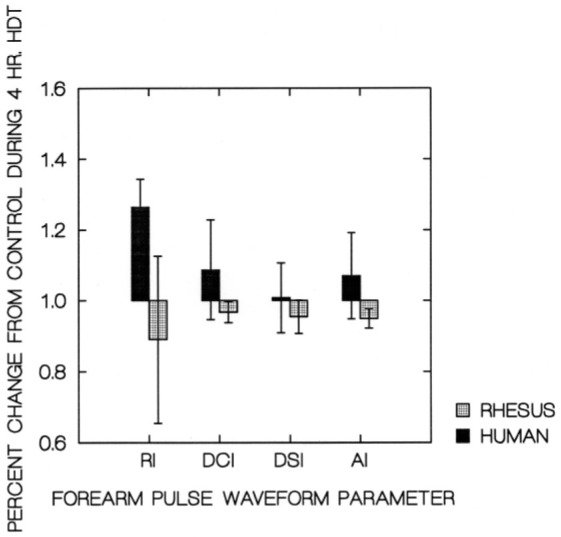
Forearm pulse waveform parameters at 4 hr HDT. No significant differences between Rhesus monkeys and humans.

Bar charts showing the segmental volumes given in Table 1, plotted relative to the control values, are presented in Figure 3. The human and Rhesus monkey calf pulse waveform parameters are plotted in Figure 4. The similar values for their forearms are plotted in Figure 5.

Table 2 lists the results of the pulsatile waveform analysis for the calf and forearm segments for the Rhesus monkeys and human subjects at 4 hours HDT.

**Table 2:** Percent calf and forearm pulse waveform parameter indices relative to control values after 4 hours of −6 degrees head down tilt.

**GROUP**	**TIME**	**NUMBER**	**RI-M**	**RI-SE**	**DCI-M**	**DCI-SE**	**DSI-M**	**DSI-SE**	**AI-M**	**AI-SE**
HUMAN-CALF	240	12	1.000	0.074	1.030	0.096	0.910	0.109	1.048	0.053
RHESUS-CALF	240	8	1.068	0.173	0.754	0.095	0.727	0.107	1.021	0.051
HUMAN-L.ARM	240	12	1.264	0.080	1.087	0.141	1.008	0.099	1.070	0.122
RHESUS-L.ARM	240	8	0.890	0.236	0.967	0.030	0.954	0.047	0.949	0.027

Segment name - M = Group mean percent volume change from control

Segment name - SE = Group standard error

## Discussion

The work described above represents the second phase of a larger study to compare the volume and circulatory responses of humans, Rhesus monkeys, and squirrel monkeys to short term orthostatic stress. The objective of the combined investigation was to determine if the squirrel monkey could be a model for Rhesus monkeys and if the Rhesus monkey could be a model for humans during various biomedical inquiries.

The first part of this work was conducted using Rhesus and squirrel monkeys during short term head up and sown tilt. The results of this initial work are described in a separate article published in the Journal of Electrical Bioimpedance 2020 (11).

Impedance plethysmography was used to measure calf, thigh, pelvic, abdominal, and thoracic volume changes in ten rhesus and eight squirrel monkeys during five minute exposures to head up tilt (HUT) and head down tilt (HDT) at angles of 5, 10, and 20 degrees. Calf, rump and tail measurements were made in three squirrel monkeys at 10 and 20 degrees of HUT and HDT. Fluid volumes in all segments of the Rhesus monkeys were found to change during HUT an HDT in direct relation to the angle of tilt used. However, the volume changes that occurred in the squirrel monkeys were found to be quite different.

Their calf, thigh, and pelvic segments lost volume during both HUT and HDT while their abdominal and thoracic segments responded similarly to those of the Rhesus monkeys.

These results and those of the calf/tail measurements of the squirrel monkeys (11) suggest that squirrel monkeys may utilize their tails as a compensatory reservoir under some postural changes and orthostatic test conditions.

To the best of our knowledge, the work described herein together with the initial article (11) represent the first direct comparison of the segmental fluid redistribution between human and primate subjects during orthostatic stress. In general, the segmental fluid responses in the human subjects are similar to those of the Rhesus monkey. However, the upper and lower arm segments of the rhesus monkeys exhibited a larger volume increase (P<0.05) during the 240 min test period than did the forearms of the human subjects.

In addition, the calf RI and DSI pulse waveform parameters of the Rhesus monkeys were quite different and in opposite direction (P<0.05) than those of the human subjects after 4 hours of HDT. The differences in RI and DSI (shown in Figure 6) may be interpreted as a reduction in venous tone in the calves of the Rhesus monkeys. This reduction in venous tone is consistent with that found by Koenig et. al. (1) during long term HDT orthostatic stress in Rhesus monkeys. A reduction of peripheral venous tone may then lead to an increased volume in the dependent segments (such as shown in Figure 3 for the Rhesus forearms) during HDT.

## Conclusion

Based upon the results of this study and that of the earlier article (11) it may be concluded that, for the most part, the Rhesus monkey may be a useful model for humans during orthostatic stress testing. However, the squirrel monkey may not be a valid model for the Rhesus monkey (and therefore for humans) during similar orthostatic tests.
